# Eating psychopathology in ballet dancers: a meta-analysis of observational studies

**DOI:** 10.1007/s40519-021-01213-5

**Published:** 2021-05-22

**Authors:** G. A. Silverii, F. Benvenuti, G. Morandin, V. Ricca, M. Monami, E. Mannucci, F. Rotella

**Affiliations:** 1 Experimental and Clinical Biomedical Sciences “Mario Serio” Department, Diabetology Unit, AOU Careggi Hospital, University of Florence, Largo Brambilla 3, 50134 Florence, Italy; 2 Department of Health Sciences, Psychiatric Unit, AOU Careggi Hospital, University of Florence, Florence, Italy

**Keywords:** Eating disorders, Eating attitude test, Eating disorder inventory, Ballet dancers, Meta-analysis

## Abstract

**Objective:**

To assess whether ballet dancers have higher eating psychopathology mean scores than the general population.

**Methods:**

Meta-analysis of cross-sectional observational studies comparing the scores of one or more of the validated eating psychopathological scales between ballet dancers and any control groups.

**Results:**

Twelve studies were included in the metanalysis. Ballet dancers had a significantly higher EAT score (12 studies retrieved, SMD 0.82 [95% CI 0.44–1.19], *p* < 0.00001, *I*^2^ = 84)]; subgroup analysis suggested a possible role of control subjects’ choice in explaining heterogeneity. Scores on the EDI subscales of Drive for Thinness, Bulimia, and Body dissatisfaction were available from four studies; Drive for Thinness was higher in ballet dancers (SMD 0.62 [0.01, 1.22]), as well as the Bulimia scale (SMD 0.38 [0.02, 0.73], *p* = 0.04) and the Body Dissatisfaction scale (SMD 0.34 [0.15, 0.53]). Data on Perfectionism, Interpersonal problems, Ineffectiveness, and Maturity fears, were available from three studies. Higher scores in Perfectionism (SMD 0.68 [0.24, 1.12] *p* = 0.02), Interpersonal problems (SMD 0.24 [0.02, 0.47], in Inefficacy, (SMD 2.18 [1.31, 3.06]) were found for ballet dancers; on the other hand, Maturity fears scores were not significantly different between ballet dancers and controls (IV-MD = 0.15 [− 0.07, 0.36]). Seven studies reported tests not performed elsewhere.

**Discussion:**

Ballet dancers show a higher level of restriction and drive for thinness than controls, and they may be, therefore, at higher risk for the development of eating disorders. Available studies do not allow the discrimination of dysfunctional eating attitudes and behaviors from adaptive responses.

**Level of evidence:**

Level I (evidence obtained from systematic reviews and meta-analyses).

**Supplementary Information:**

The online version contains supplementary material available at 10.1007/s40519-021-01213-5.

## Introduction

Eating disorders (ED), a heterogeneous group of disorders encompassing Anorexia Nervosa (AN), Bulimia Nervosa (BN), Binge Eating Disorder (BED) and Eating Disorders Not Otherwise Specified (EDNOS), are more prevalent in female adolescents, which have a 10% lifetime risk of clinical ED [1]; on the other hand, the subclinical disorders’ rate remains unclear [1]. It has been estimated that only one in four adolescents with ED receives an appropriate treatment [2]. Identifying groups at higher risk for ED in the general population is crucial for a better understanding of risk factors for ED, as well as for developing possible prevention strategies [3] in specific high-risk groups of adolescents [4], such as ballet dancers [5–8]. Low self-esteem and perfectionism may play a dominant role in the development and maintenance of dysfunctional eating behaviors in ballet dancers [9]. Drive to thinness, hard discipline, high personal standards and body perfectionism, which are frequent in ballet dancing, may all contribute to dysfunctional eating behaviors [3]. In clinical practice and research, eating psychopathological traits are often addressed using psychometric scales, i.e. self-reported questionnaires or structured or semi-structured clinical interviews; all these tools are mostly made up by Likert-type response items [10], and explore disorders’ presence and gravity in a dimensional way to point out patients’ attitudes towards dieting, shape and body concerns, strategies for controlling eating behaviors, and somatic symptoms [11].

Body dissatisfaction, self-objectification and thin-ideal internalization seem to be the main factors that further increase the risk for development of eating disorders [12, 13], and body dissatisfaction seems to be one of the most important risk factor in the maintenance of these pathologies [14]. Some studies have pointed out that ballet dancers, even when not fulfilling diagnostic criteria for EDs, have body dissatisfaction and drive for thinness scores comparable to those of patients suffering from EDs [15]. Some psychometric scales, such as the Eating Attitude Test (EAT), have been used to recognize subjects at high risk for eating disorders [16, 17].

A meta-analysis of cross-sectional studies performed a few years ago [18] reported an increased overall prevalence of eating disorders in ballet dancers in comparison with matched control groups. When exploring the different diagnostic categories within ED, significant differences between ballet dancers and controls were observed for EDNOS, but not for any other diagnostic category. This finding has limited clinical usefulness, as it does not allow to discriminate whether ballet dancers are at higher risk of AN, or BN, or BED, and it may depend on the limited size of available samples. In addition, the previously cited meta-analysis [18] does not provide meta-analytic estimates of differences in scores of psychometric tests, summarizing only categorical diagnoses. A dimensional approach, focused on the results of psychometric tests rather than on categorical diagnoses, could have a greater statistical power, allowing the observation of otherwise undetected features of ballet dancers in different areas of eating disorder psychopathology. For this reason, it seems important not only take into account categorical criteria but also to focus on psychopathological dimensions.

The aim of our study was to assess whether ballet dancers have higher eating psychopathology scores than the general population.

## Methods

This meta-analysis is reported following the criteria of PRISMA statement [19]. The review protocol was registered on the University of York Centre for Reviews and Dissemination Prospero Web site (registration number: CRD42020185945) [20]. This is a meta-analysis study, and it is therefore exempt from ethics approval, as the study authors only collected and synthesized all-anonymised data from previous clinical trials, in which informed consent has already been obtained by the trial investigators.

### Search strategy and selection criteria

A systematic research on PubMed and Embase databases was performed, collecting all observational studies on eating attitudes and behaviors comparing ballet dancers to control subjects, with no age restriction up to September 1st, 2020.

Titles and abstracts of studies were retrieved from MEDLINE and EMBASE.

The MEDLINE search strategy was the following: ("feeding and eating disorders"[MeSH Terms] OR ("feeding"[All Fields] AND "eating"[All Fields] AND "disorders"[All Fields]) OR "feeding and eating disorders"[All Fields] OR ("eating"[All Fields] AND "disorders"[All Fields]) OR "eating disorders"[All Fields]) AND ("dancing"[MeSH Terms] OR "dancing"[All Fields] OR "ballet"[All Fields]).

The EMBASE search strategy was the following: “eating AND disorders AND ballet AND [Embase]/lim NOT ([Embase]/lim AND [MEDLINE]/lim).

Further studies were searched among references from papers retrieved through database search.

Studies were included if they fulfilled the following criteria:

1. Observational studies comparing the prevalence of eating behavior between ballet dancers and any control groups.
2. Studies reporting scores of one or more of the following psychopathological scales:
  The Eating Attitude Test, either EAT-40 [21] (Garner and Garfinkel1979) or EAT-26 [16], exploring shape perception, bulimia and food concern and oral control [16].
  The Eating Disorders Inventory (EDI) [22]. In its latest version (EDI-3), this 91-item questionnaire explores 12 domains: Drive for thinness (DT), Bulimia (B), Body dissatisfaction (BD), Low self-esteem (LSE), Personal alienation (PA), Interpersonal insecurity (II), Interpersonal alienation (IA), Interoceptive deficits (ID), Emotional dysregulation (ED), Perfectionism (P), Asceticism (AS), Maturity fears (MF) [22].
  The Eating Disorders Examination, either as interview (EDE; [23, 24]) or Questionnaire (EDE-Q) [25], which is composed by four domains: Restraint (R), Eating Concern (EC), Shape Concern (SC), and Weight Concern (WC).
  The Low Energy Availability in Females Questionnaire (LEAF-Q), a brief ED screening questionnaire which investigates the risk of the Triad (ED, amenorrhea, osteoporosis) in female athletes, through three main domains: Injuries in the last year, gastrointestinal function and menstrual function [26].
  The Silhouette Test for Adolescents (STA): composed by a series of human body figures, it helps to describe the risk of development of ED by analyzing the body image distortion and corporal dissatisfaction rates [27].
  The Body Uneasiness Test (BUT), a 71-item self-report questionnaire which could be useful to screen abnormal body image attitudes. It is composed by two parts (BUT-A and BUT-B): first one investigates weight phobia (WP), body image concerns (BIC), avoidance (AVO), compulsive self-monitoring (CSM), detachment and depersonalization (D); the second one aims to point out main worries about particular body parts or functions [28].
  The Bulimic Investigatory Test, Edinburgh (BITE), which describes eating disorders patterns and binge eating behavior by a 33-item self-report measure questionnaire. Three main classes can be identify through the result scores: high-risk group (> 20), medium-risk group (10–19) and low-risk’s one (< 10) [29].
  The Semi-quantitative Food Frequency Questionnaire (SFFQ), a simple useful questionnaire that explores and quantify individual dietary intake [30].
  Any other Questionnaire or Semi-structured interview exploring eating psychopathology.
3. Studies written in English, French, Spanish or Italian language.

Studies were excluded if they enrolled both ballet dancers and other categories of dancers without providing subgroup analyses.

### Data analysis and synthesis

Retrieved titles and abstracts were screened independently by two review team members (F.B. and G.M.) to identify studies potentially meeting the inclusion criteria outlined above. The full text of these potentially eligible studies was retrieved and independently assessed for eligibility. Any disagreement between over the eligibility of particular studies was resolved through discussion with a third reviewer (GA.S.).

A standardized, pre-piloted form was used to extract data from the included studies for assessment of study quality and evidence synthesis. The following parameters/information were extracted from included articles: first author, year of publication; number of subjects enrolled in each arm; mean age, body mass index and proportion of males and females in each arm; features of the control groups; study setting; study population; recruitment and study completion rates; outcomes; information for assessment of the risk of bias.

### Risk of bias (quality) assessment

Two review authors independently assessed the risk of bias in included studies using The Newcastle–Ottawa Scale (NOS) [31] for assessing the quality of non-randomized studies in meta-analyses. Disagreements between the review authors over the risk of bias in particular studies will be resolved by discussion, with involvement of a third review author where necessary (GA.S. and M.M.), and conflicts were resolved through discussion with a third investigator (F.R).

### Statistical analyses

The endpoints were the differences in mean scores of each psychopathological scale and subscale, between ballet dancers and any control group, either athletes or non-athletes. Aggregate study data were used for a quantitative synthesis. Between-group standardized difference in means (standardized mean difference: SMD), in the Hedges’ (adjusted) *g* variant, with 95%, CI were calculated. Heterogeneity was assessed by using *I*^2^ statistics. In case of significant heterogeneity, subgroup analyses and meta-regressions were performed, exploring the possible effects of age and gender of enrolled subjects, Country in which the study was performed, and strategy for identification of control subjects. A random-effects model was applied as the primary analysis; fixed effect model was used as a sensitivity analysis. Egger’s regression was performed to estimate possible publication/disclosure bias; within-group reliability coefficient was adopted to correct for possible measurement error [32, 33]. All analyses were performed using Review Manager 5.3.5; The Cochrane Collaboration, 2014, and Comprehensive Metanalysis (Biostat Inc. 14 North Dean Street Englewood, NJ 07631 USA).

## Results

Out of 175 studies identified, 18 studies fulfilling the inclusion criteria specified above were retrieved; of those, 12 could be included in Metanalysis, enrolling a total of 577 ballet dancers and 728 controls. The search flow is illustrated in Fig. 1S of supplementary materials, whereas the main characteristics of the selected studies, as well as risk of bias and study quality, assessed through the Newcastle–Ottawa scale, are reported in Table 1. Of the 18 studies, ten were performed in Europe, six in North-America, one in South America and one in Australia.

**Table 1:** Characteristics of the studies

Study	CO	NOS	Ballet dancers						Scale	Controls						
			*N*	*M*	AM	Age	BMI < 17	BMI		Category	*N*	*M*	AM	Age	BMI < 17	BMI
Abraham 1996 [9]	AU	5	60	nr	58.3	16.9	21	nr	EAT-40	Students	216	nr	nr	16.1	17	nr
Bettle 1998 [43]	DE	4	57	35.1	nr	14	nr	F 16.8 M 16.6	EAT-40	Students	154	39.1		15.2	Nr	F19.8 M19.5
Bettle 2001 [8]	DE	5	90	35.6	nr	13.5	nr	nr	SDTQ	Students	156	39.0	Nr	13.5	Nr	nr
Braisted 1985 [6]	US	4	45	0	6.7	16 .1	Nr	16.73	SSI	Volunteers	44	0	0	14.7	Nr	18.96
Frusztajer 1990 [44]	US	4	20	0	50	20.5	13.3	18.58	EAT-26	Non-dancers	10	0	40	20.5	10.1	19.27
Garner 1980 [4]	CA	4	183	0	28	18.6	nr	18.7	EAT-26	Anorexia Nervosa Normal controls Modeling students Music students	68 81 56 35	0	NR	23.2 21.4 21.5 15.2	14.7	16.7 22.18 18.83 19.91
Herbrich 2011 [45]	DE	5	52	0	25	16.4	44.3	18.2	EDI-2 MSCS	Non-athletic students Anorexia Nervosa	44 52	0	0 100	16.7 15.8	NR	20.0 15.2
Holderness 1994 [46]	US	4	50	0	36	21		19.16	EAT-26	Non-dancers	56	0	36	22.87	13.79	20.47
Kaufman 2002 [47]	US	4	21	0	23.8	23.2	NR	19.5	EAT-26	Healthy controls	27	0	4	24.5	Nr	19.8
Diogo 2016 [41]	BR	3	92	Nr	Nr	Nr	Nr	Nr	EAT-26	Jazz/street dancers	130	Nr	Nr	Nr	Nr	Nr
Martin 1989 [7]	FR	3	23	0		18.3	51.2	18	EAT-40	Healthy controls	23	0		26.4	12	20.4
Monthuy-Blanc 2010 [46]	FR	4	50	0	NR	NR	15.4	19.2	EDI-1	Non-athletes Athletes	47 95	0	NR	14.1 14.1	6.1 13.7	19.2 19.3
Neumärker 1998 [34]	DE	4	57	35.1	Nr	15.1	Nr	Nr	EAT-26 EDI-1	Students	156	39.1	Nr	17.4	Nr	Nr
Ravaldi 2006 [48]	IT	4	110	0		16.5	1.6	19	EDE T	Non-athletic students	59	0		26.12	1.7	19.5
Ringham 2006 [15]	US	4	28	0		19.66	NR	20.9	EDI-2	Healthy AN	44 25	0	EDI-2	24.5 26.1		22.4
Tolgyes 2004 [35]	HU	4	33	39.4		15.5	Nr	Nr	EAT 40 BITE	Students	33	45.5	Nr	14	Nr	Nr
Kazarez 2018 [36]	ES	4	100	0	Nr	15.41	0.80	19.72	STA-DI	Contemporary dancers Spanish dancers	198	0	Nr	16.2 16.3	0.26	20.71 21.21
Weeda-Mannak 1985 [37]	NL	4	105	0	nr	19.4	NR	19.8	ANIS	AN Healthy Controls	84 237	0	nrr	24.7 19.4	Nr	17.81 21.11

*CO* Country in which the study was performed. *Nr* Not reported; *AN* Anorexia Nervosa; *BN* Bulimia Nervosa; *N* Number; *M* Males (%); *NOS* Newcastle–Ottawa Scale (risk for bias); *SDTQ* Semantical Differential Technique; *U* Unattractiveness; *B-M* Body-Mass; *P* Purity; *UD* Undesirability; *S* Sensitivity; *SSI* Semi-structured Interview; *BITE* Questionnaires Bulimic Investigatory Test Edinburgh; *BUT* Body Uneasiness Test; *WP* weight phobia; *BIC* body image concerns; *AVO* avoidance; *CSM* compulsive self-monitoring; *D* detachment and depersonalization; *GSI* Global Severity Index; *PST* positive symptoms total; *PSDI* Positive Symptom Distress Index; *STA-DI* Silhouette test for adolescents-Distortion Index; *MSCS* Multidimensional Self-Concept Scale; *EAT* Eating Attitude Test—The Eating Disorders Examination, EDE Restraint (R), Eating Concern (EC), Shape Concern (SC), and Weight Concern (WC); *ANIS* anorexia nervosa inventory for self-rating; *AM* Amenorrhea

Four studies provided a comparison between ballet dancers and unspecified healthy controls; in two studies, there were both unspecified controls and Anorexia Nervosa (AN) patients, whereas two studies compared ballet dancers with different groups of dancers, and seven with other students. Only two studies of those included in the meta-analysis [34, 35] enrolled male subjects, and they both provided subgroup results for males and females; therefore, males and females were considered as two different subgroups.

### Eating Attitude Test (EAT)

**Fig. 1:**
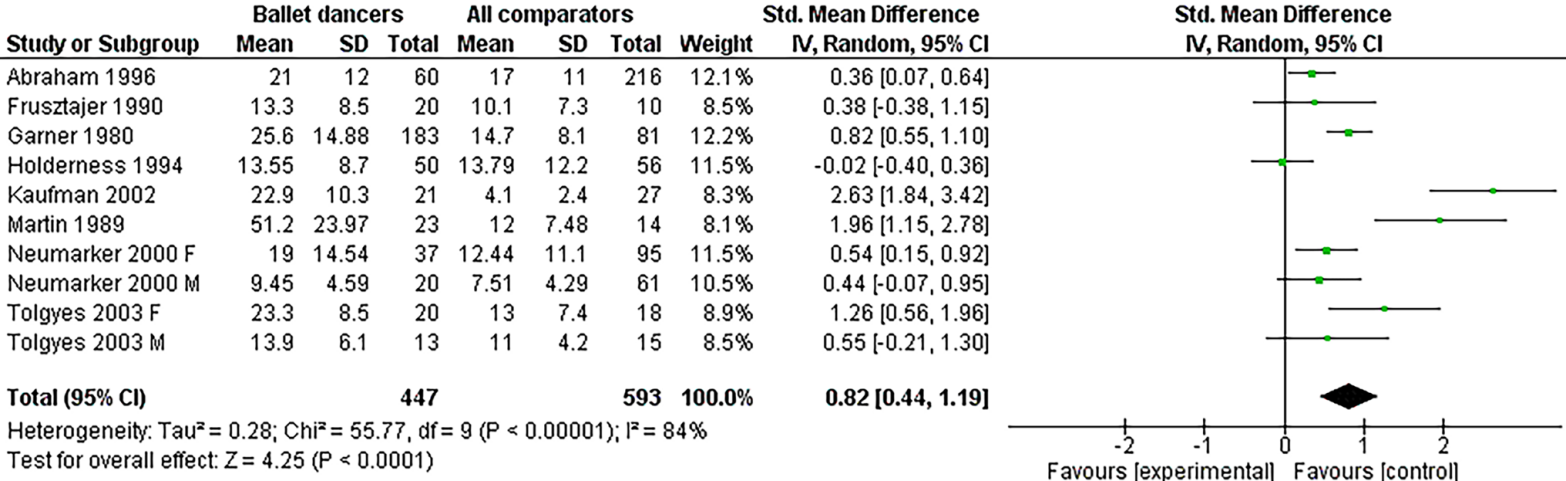
Standardized mean difference in means for EAT scale scores. *STD mean difference* Standardized difference in means, *IV* Inverse Variance, *95% CI* 95% Confidence Interval

Several questionnaires were adopted to describe the prevalence of eating psychopathology (Tables 1 and 2). Twelve studies used the EAT-26 or EAT-40 scales, which we both pooled in a unique analysis, using standardized difference in means (Fig. 1). The Egger’s regression test did not suggest significant publication bias (*I* = 3; *p* = 0.14). Ballet dancers had a significantly higher EAT score (SMD 0.82 [95% CI 0.44–1.19], *p* < 0.00001 for overall effect). When correcting for the EAT 40 and EAT 25 reliability index [16, 21], the SMD retained significance (SMD 0.86 [0.46–1.23]) the unstandardized mean difference was in EAT 40 was 9.15 points, and 7.27 points for EAT 26. Sensitivity analysis were performed, using a fixed effect model, which confirmed the outcome (SMD 0.62 [95% CI 0.48–0.76], as well as analyses performed excluding one of the studies (Table 1S of supplementary materials). Heterogeneity was high (*I*^2^ = 84%), and remained such even when excluding males (*I*^2^ = 88%); in females only, SMD was 0.74 [0.56, 0.91]. No significant difference between groups was shown when performing meta-regression analyses accounting for mean age of participants (*p* = 0.62), year of publication (*p* = 0.23), or NOS score (*p* = 0.45); on the other hand, the age of controls was directly related to difference in EAT (*r* = 0.05 [0.01–0.2], *p* = 0.012) (Table 2S of supplementary materials). A subgroup analysis found no difference for country of origin (*p* = 0.19 for European or American). On the other hand, when dividing for type of control subjects, the studies which specifically chose non-athlete controls (normal controls) had a higher standardized difference in means than studies which randomly chose controls subjects (1.76 [0.56–2.96] vs. 0.43 [0.18–0.68]; difference between subgroups was significant (*p* < 0.0001), again suggesting a possible role of control subjects’ choice in explaining heterogeneity (Fig. 2S of supplementary materials).

**Table 2:** Eating psychopathology scores for ballet dancers and controls in the included studies

Study	BD	Scale	Controls	AN
Abraham 1996 [9]	21	EAT-40	17	
Bettle 2001 [8]	17.95 16.59	SDTQ-U SDTQ–P	16.64 16.3	
	24.23	SDTQ-S	22.63	
	19.69	SDTQ-BM	20.9	
	28.75	SDTQ-UD	26.01	
Frusztajer 1990 [44]	13.3	EAT-26	10.1	
Garner 1980 [4]	25.6	EAT-26	14.7	58.3
Herbrich 2011 [45]	23.58	EDI-2-I	20.55	33.85
	16.52	EDI-2-DT	20.58	28.69
	23.79	EDI-2-IA	20.20	28.98
	23.10	EDI-2-IR	19.73	24.60
	20.67	EDI-2P	18.32	19.60
	14.06	EDI-2B	11.11	11.40
	24.65	EDI-2-MF	23.57	26.88
	21.06	EDI-2-SI	19.18	27.60
	18.50	EDI-2-ID	16.52	22.58
	29.63	EDI-2-BD	26.89	37.65
	18.54	EDI-2-A	15.82	23.02
Holderness 1994 [46]	13.55	EAT-26	13.79	
Kaufman 2002 [47]	22.9	EAT-26	21	
Martin 1989 [7]	51.2	EAT-40	12	
Monthuy-Blanc 2010 [49]	1.82	EDI -2- B	2.28	
	7.6	EDI-2-DT	4.77	
	10.28	EDI-2-BD	8.32	
Neumärker 1998 [34]	15.64	EAT-26	10.86	
	F 6.4 m0.6	EDI-2 DT	F3.88 M1.15	
	F1.46 M0.75	EDI-2 B	F0.91M0.62	
	F 10.19 M3.55	EDI-2 BD	F 8.56 M2.23	
	F3.14 M1.7	EDI-2 A	F2.65 M1.3	
	F4.78 M2.75	EDI-2 I	F3.33M1.31	
	F 6.05 M 5.9	EDI-2-MF	M 6.72 F 5.87	
	F 4.33 M4.85	EDI-2 P	M 4 F 2.01	
	F 4 M 2.85	EDI-2 ID	F2.01 M3.2	
Ravaldi 2006 [48]	1.6	EDE T	1.7	
	2.1	EDE WC	2.1	
	0.8	EDE R	1.6	
	2.7	EDE SC	2.8	
	0.4	EDE EC	0.2	
	2.3	BUT PSDI	2.1	
	1.5	BUT CSM	0.8	
	2.0	BUT WP	1.2	
	0.2	BUT D	0	
	1.2	BUT BIC	1.1	
	13.8	BUT PST	9.2	
	1.1	BUT GSI	0.6	
	1.2	BUT AVO	1.1	
Ringham 2006 [15]	11.27	EDI-2 DT	1.00	9.16
	2.32	EDI-2 B	0.23	0.56
	12.14	EDI-2 BD	7.14	11.12
	5.18	EDI-2 A	0.89	3.56
	4.18	EDI-2 I	1.11	5.76
	2.72	EDI-2-MF	1.80	4.6
	7.05	EDI-2 P	2.80	8.16
	2.50	EDI-2 ID	1.45	4.16
Tolgyes 2004 [35]	23.3	EAT 40	13	
	13.8	BITE	7.2	
Kazarez 2018 [36]	0.80	STA-DI	0.26	
Weeda-Mannak 1985 [37]	106	ANIS	97.3	143

*AN* Anorexia Nervosa; *U* Unattractiveness; *B-M* Body-Mass; *P* Purity; *UD* Undesirability; *S* Sensitivity; *BITE* Questionnaires Bulimic Investigatory Test Edinburgh; *BUT* Body Uneasiness Test; *WP* weight phobia; *BIC* body image concerns; *AVO* avoidance; *CSM* compulsive self-monitoring; *D* detachment and depersonalization; *GSI* Global Severity Index; *PST* positive symptoms total; *PSDI* Positive Symptom Distress Index; *STA-DI* Silhouette test for adolescents-Distortion Index; *EAT* Eating Attitude Test; *EDE* Eating Disorders Examination; *R* Restraint; *EC* Eating Concern; *SC* Shape Concern; *WC* Weight Concern; *ANIS* anorexia nervosa inventory for self-rating; *DT* Drive for Thinness; *B* Bulimia; *BD* Body Dissatisfaction; *I* Ineffectivity; *P* Perfectionism; *ID* Interpersonal Distrust; *IA* Interoceptive Awareness; *MF* Maturity Fears; *A* Asceticism; *IR* Impulse Regulation; *SI* Social Insecurity

### Other tests

Scores on the Eating Disorder Inventory (EDI) subscales were available from four studies (Table 2). Drive for Thinness, Bulimia, Body Dissatisfaction, Interpersonal problems, Perfectionism and Ineffectiveness were higher in ballet dancers, whereas maturity fears scores were not significantly different between ballet dancers and controls (Table 2).

One Spanish study [36] adopted the Silhouette Test for Adolescents (STA) to analyze differences in body shape distortion between 100 ballet dancers, 75 contemporary dancers and 123 Spanish dancers. Ballet dancers were younger and had a lower BMI. Higher values of distortion index, which describes the gap between perceived and real body image, were found in ballet dancers versus contemporary (*p* = 0.002) and Spanish dancers (*p* < 0.001).

Tölgyes et al. [35] adopted the Bulimic Investigatory Test Edinburgh (BITE); they found higher scores in secondary school ballet students (*n* = 20 females, 13 males) in comparison to non-dancer students (*n* = 16 females and 17 males).

In one study [37], 105 female ballet dancers showed significantly higher Anorexia Nervosa Inventory of self-rating (ANIS) [38] scores than 237 normal controls; however, their scores were lower than those of 84 women with AN.

Braisted et al. enrolled 45 ballet dancers (BD) and 44 female volunteers (C), and they found a significantly higher prevalence of distortion of body image in ballet dancers than in the control group [39].

Two further studies by Ravaldi et al. [40, 48] reported higher body dissatisfaction, measured with the Body Uneasiness Test (BUT), and eating disorder psychopathology, assessed through the Eating Disorders Examination questionnaire (EDE-Q), in ballet dancers, compared with different controls (female gymnasium users, male non-competitive body builders, subject not performing sports, and unselected female students).

A Brazilian study [41] showed that ballet dancers had a higher proportion of subjects with EAT-26 score ≥ 20, meaning a higher risk of presenting eating disorders, whereas jazz practitioners had a lower risk. Bettle et al. [34] enrolled 58 female and 32 male ballet students (11–18 ys) and a control group composed by 95 female and 61 male students (13–18 ys), EAT-40 EAT score > 30 was found in 8 female ballet dancers, and in none of the control group. In a subsequent study [8] in which questionnaires built on semantic differential technique were adopted, they found significant differences between female ballet dancers and controls on unattractiveness for body, undesirability and sensitivity for personality. A higher unattractiveness of body and undesirability were described between younger male dancers and younger female dancers.

## Discussion

Our meta-analysis shows a higher prevalence of eating psychopathology in ballet dancers, when compared to controls. To our knowledge, this is the first meta-analysis summarizing scores of psychometric tests on eating attitudes and behavior from controlled studies on ballet dancers. Our results suggest that ballet dancers show higher levels of restriction and drive for thinness than those of controls.

The higher levels of restriction and drive for thinness among ballet dancers are consistent with a higher prevalence of perfectionism; in fact, maintaining thinness could play an adaptive role in the achievement of performance in ballet. It has been reported that high-performance sports, in which body shape plays a relevant role (such as gymnastic, dance, skating, swimming and running) [5] all require increased levels of perfectionism, self-control and eating control. In this framework, distinguishing the adaptive could lead to a better therapeutic approach. A clinical-oriented approach in adaptive behavior context may have a potentially detrimental impact on ballet career, without any health benefit. However, evaluations such as the EAT and the EDI questionnaires, which are based on ballet dancers’ belief and self-perception, may have limited reliability in assessing the possible disruptive effect of these dimensions by the egosyntonic o egodistonic ballet dancers’ believe, because of the well-known absence of patients’ insight. Therefore, other psychometric tests which investigate anxiety (such as Quality Of Life, State-Trait Anxiety Inventory), or psychopathological scales focused on body image distortion, as the Body Uneasiness Test, which have been used only in few studies, may provide greater accuracy in detecting eating psychopathology. Moreover, a follow-up re-evaluation of eating psychopathology may be needed, when performing is no longer requested.

Two studies compared ballet dancers with anorexia patients, both showing that ballet dancers’ features may be considered intermediate between the normal and anorexia patients. In this framework, it should be noted that a very two prospective studies, showed that most ballet dancers showing signs of ED at baseline improved spontaneously [42] with a high rate of spontaneous weight gain [39]. These data seem to confirm that eating restriction in ballet dancers could be a peculiar feature, and be considered, in some dancers at least, functional to a successful adaptation, rather than dysfunctional [7].

Some limitations of our meta-analysis have to be mentioned: the number of eligible studies was small, and sample sizes were limited. In addition, some of the studies showed methodological limitations, mainly because of suboptimal matching of cases and controls for age and socio-demographic characteristics; this may explain the heterogeneity of most results. Moreover, different studies were performed using different questionnaires, posing a further challenge to meta-analyses.

In conclusion, ballet dancers show a higher level of restriction and drive for thinness than control subjects. This result suggests that ballet dancers could be a group at risk for the development of eating disorders, and anorexia nervosa in particular. Available studies do not allow the discrimination of dysfunctional eating attitudes and behaviors from adaptive responses to the requirement of thinness for performance in ballet. Further studies, including assessments of quality of life and of related psychopathology, and longitudinal observations, are needed for a better characterization of eating psychopathology in ballet dancers. A better understanding of the impact and prognosis of disturbed eating attitudes is essential for the definition of therapeutic interventions, thus deserving further investigation.

## Strength and limits

The strength of this study was his dimensional rather than categorical approach. Limitations were a small number of eligible studies, some of which showing methodological limitations, and small sample size. Considering the limited number of available studies, the assessment of publication bias cannot be considered fully reliable.

## What is already known on this subject?

The overall prevalence of eating disorders is increased in ballet dancers in comparison with matched controls, but no significant difference was found for any category within ED, except for EDNOS.

## What this study adds?

We summarize psychometric scores on eating attitudes from controlled studies on ballet dancers, suggesting that they show higher levels of restriction and drive for thinness than controls.

## Supplementary Information

Below is the link to the electronic supplementary material.

Supplementary file1 (DOCX 78 KB)

## Data Availability

The corresponding author had full access to all the data in the study and takes responsibility for the integrity of the data and the accuracy of the data analysis. Data sharing is not applicable to this article as no new data were created or analyzed in this study.
