# Supplementary material for: Eating psychopathology in ballet dancers: a meta-analysis of observational studies
Source: Eat Weight Disord. 2021 May 22;27(2):405–14. doi: 10.1007/s40519-021-01213-5 (PMC8933308; doi:10.1007/s40519-021-01213-5)
Supplement: Supplementary file 1 — Supplementary file1 (DOCX 78 KB) [file 40519_2021_1213_MOESM1_ESM.docx]

**Eating psychopathology in ballet dancers: a meta-analysis of observational studies. Supplementary materials**


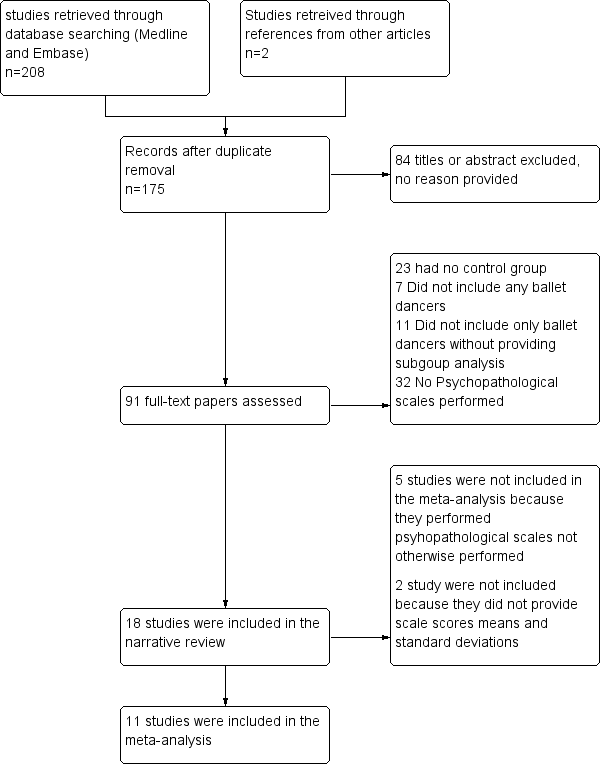


Fig.1S: study research flow

| Study excluded | P for effect | SMD |
| --- | --- | --- |
| Abraham 1996 | P<0.0001 | 0.89 [0.45, 1.33] |
| Frusztajer 1990 | P<0.0001 | 0.86 [0.46, 1.26] |
| Garner 1980 | P=0.0003 | 0.83 [0.38, 1.28] |
| Holderness 1994 | P<0.00001 | 0.92 [0.53, 1.31] |
| Kaufman 2002 | P<0.0001 | 0.63 [0.33, 0.93] |
| Martin 1989 | P=0.0001 | 0.71 [0.34, 1.07] |
| Neumarker 2000 | P=0.0002 | 0.93 [0.44, 1.41] |
| Tolgyes 2003 | P<0.00001 | 0.80 [0.37, 1.23] |

Tab. 1S: Sensitivity analysis: standardized difference in means in EAT between ballet dancers and controls when excluding one study (in the left column) from analysis


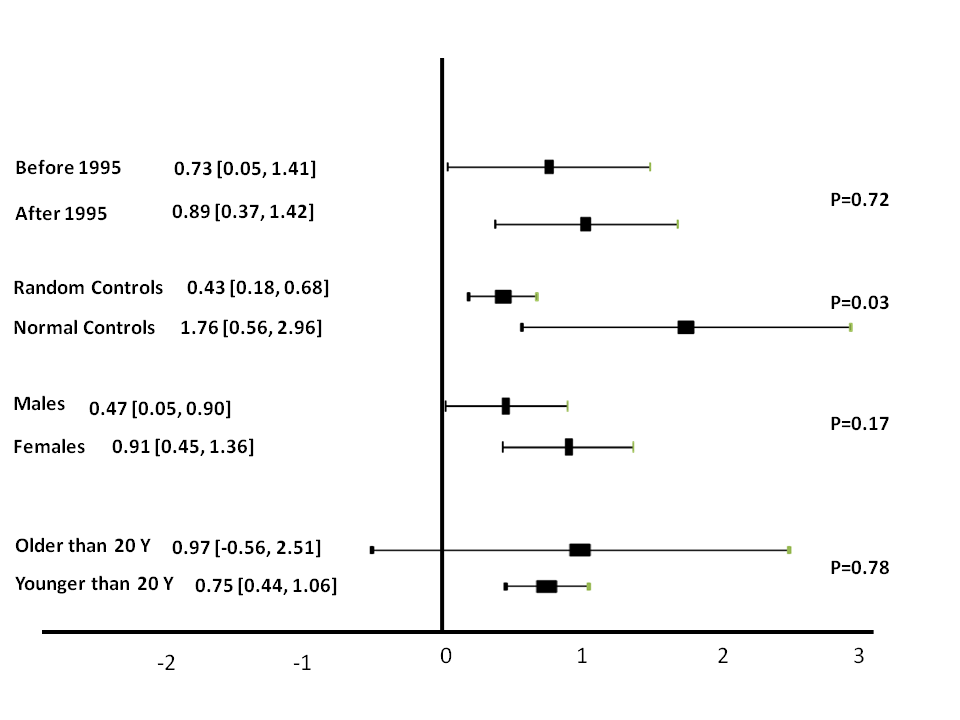


| Moderator | r | P |
| --- | --- | --- |
| Age of Ballet Dancers | 0.004 [-0.013, 0.022] | P=0.62 |
| Age of controls | 0.05 [0.01, 0.09] | P=0.014 |
| NOS score | 0.07 [-0.11, 0.24] | P=0.46 |
| Year of publication | -0.006 [-0.024, 0.011] | P=0.49 |

Fig. 2S Metaregression analysis: subgroup analysis for in standardized difference in means in EAT between ballet dancers and controls.

Tab. 2S Metaregression analysis: effect of different moderators on difference in in standardized difference in means in EAT between ballet dancers and controls

Fig. 3S Metaregression analysis: effect of age of controls in standardized difference in means in EAT between ballet dancers and controls
